# Supplementary material for: Automated cot‐side tracking of functional brain age in preterm infants
Source: Ann Clin Transl Neurol. 2020 May 5;7(6):891–902. doi: 10.1002/acn3.51043 (PMC7318094; doi:10.1002/acn3.51043)
Supplement: Supplementary file 1 — Table S1: Correlation between functional brain age estimates, based on a single qEEG variable, and post‐menstrual age (PMA) and variable selection frequency for multivariable models of FBA. [file ACN3-7-891-s001.pdf]

Table S1: Correlation between functional brain age (FBA) estimates, based on a single qEEG variable, and post-menstrual age (PMA) and variable selection frequency for multivariable models of FBA. All results were estimated within a leave-one-subject-out cross-validation. Type defines: B - a set representing measures of bursts, P - a set representing phenomenological interpretation of the EEG, O - a set representing other measures in the literature, [1] denotes bursts defined according to [1], [2] denote bursts defined according to [2], and selected denotes variable selection frequency.

rank	qEEG variable	type	r	selected
1	Asymmetry (all)	B	0.874	29
2	Asymmetry (500ms-1s)	B	0.851	64
3	Sharpness (500ms-1s)	B	0.845	51
4	Burst Number[1] (4-8s)	B	0.843	45
5	Suppression Curve	O	0.842	63
6	Burst Number[1] (2-4s)	B	0.842	65
7	Asymmetry (250-500ms)	B	0.829	35
8	mean Phase Locking Index	O	0.822	62
9	Path length (coherence)	O	0.811	65
10	Burst Number[1] (1-2s)	B	0.807	29
11	Multi-Scale Entropy (Δ 5 scales)	O	0.807	64
12	Multi-Scale Entropy (mean)	O	0.794	65
13	Multi-Scale Entropy (maximum)	O	0.778	60
14	Mean Burst Duration[1]	B	0.777	24
15	Burst Number[1] (125-250ms)	B	0.772	64
16	Sharpness (1-2s)	B	0.720	57
17	Burst Number[1] (500ms-1s)	B	0.713	28
18	Asymmetry (1s-2s)	B	0.676	28
19	Slope of linear fit to burst area vs burst duration[1]	B	0.667	63
20	Number of bursts[1]	B	0.656	65
21	Alpha (truncated power law fit to CDF of burst size)	B	0.644	53
22	Intercept of linear fit to asymmetry vs burst duration[1]	B	0.643	35
23	Sharpness (62.5-125ms)	B	0.630	65
24	Activity Synchrony Index (global)	O	0.627	1
25	Sharpness (1-2s)	B	0.618	49
26	Range EEG (5th percentile, quiet sleep)	P	0.595	2
27	Range EEG (5th percentile, full recording)	P	0.592	47
28	Cross-Channel Correlation (full recording)	P	0.572	58
29	Inter-Burst Interval[2] (95th percentile, full recording)	P	0.572	13
30	Inter-Burst Interval[2] (median, quiet sleep)	P	0.567	1
31	Cross-Channel Correlation (quiet sleep)	P	0.566	24
32	Alpha (truncated power law fit to CDF of burst duration)	B	0.565	12
33	Sharpness (s)	B	0.563	7
34	Inter-Burst Interval[2] (median, full recording)	P	0.553	65
35	Bursts per hour[2] (full recording)	P	0.535	28
36	RMS Inter-Burst Interval[2] (full recording)	P	0.530	5
37	Bursts per hour[2] (quiet sleep)	P	0.527	6
38	Inter-Burst Interval[2] (95th percentile, quiet sleep)	P	0.525	3
39	Range EEG (95th percentile, full recording)	P	0.524	54
40	RMS Inter-Burst Interval[2] (quiet sleep)	P	0.522	1
41	Lambda (truncated power law fit to CDF of burst size)	B	0.521	19
42	Sharpness (125-250ms)	B	0.505	45
43	Intercept of linear fit to burst size vs burst duration[1]	B	0.502	65
44	EEG envelope (95th percentile, full recording)	P	0.495	57
45	Relative Alpha Power (quiet sleep)	P	0.481	65
46	Burst Number[1] (62.5-125ms)	B	0.480	37
47	EEG envelope (5th percentile, quiet sleep)	P	0.478	1
48	Activity Synchrony Index (hemispheric)	O	0.474	28
49	Log-Likelihood Ratio of Fit (truncated power law fit to CDF of burst size)	B	0.473	8
50	Sharpness (all)	B	0.467	59
51	Range EEG (95th percentile, quiet sleep)	P	0.460	42
52	Relative alpha power (full recording)	P	0.440	63

53	Inter-Burst Interval[2] (5th percentile, full recording)	P	0.440	29
54	Inter-Burst Interval[2] (5th percentile, quiet sleep)	P	0.433	10
55	Total spectral power (full recording)	P	0.433	26
56	EEG envelope (5th percentile, full recording)	P	0.425	33
57	Temporal Theta Power (full recording)	P	0.425	44
58	EEG Envelope (95th percentile, quiet sleep)	P	0.416	16
59	Asymmetry (2-4s)	B	0.353	24
60	Log-Likelihood Ratio of Fit (truncated power law fit to CDF of burst duration)	B	0.341	60
61	Total spectral power (quiet sleep)	P	0.338	35
62	Burst duration[2] (median, quiet sleep)	P	0.337	17
63	Temporal Theta Power (quiet sleep)	P	0.328	47
64	Asymmetry (125-250ms)	B	0.32	29
65	Burst duration (5th percentile, quiet sleep)	P	0.324	33
66	s_{min} (truncated power law fit to CDF of burst duration)	B	0.323	15
67	Burst duration[2] (5th percentile, full recording)	P	0.304	16
68	Activity Synchrony Index (quiet sleep)	P	0.283	0
69	Segment Rate	O	0.246	36
70	Activity Synchrony Index (full recording)	P	0.245	65
71	Coefficient of variation (burst durations)[1]	B	0.231	61
72	Slope of linear fit to sharpness vs burst duration[1]	B	0.227	19
73	Burst duration[2] (median, full recording)	P	0.227	3
74	Relative delta 1 power (quiet sleep)	P	0.221	58
75	Relative delta 2 power (quiet sleep)	P	0.210	23
76	Range EEG (median, quiet sleep)	P	0.206	14
77	Lambda (truncated power law fit to CDF of burst duration)	B	0.202	45
78	Burst duration[2] (95th percentile, full recording)	P	0.193	10
79	EEG Envelope (median, quiet sleep)	P	0.187	2
80	RMS burst duration[2] (full recording)	P	0.169	2
81	Relative delta 2 power (full recording)	P	0.166	65
82	Relative delta 1 power (full recording)	P	0.151	60
83	Asymmetry (4-8s)	B	0.130	14
84	Slope of linear fit to asymmetry vs burst duration[1]	B	0.120	6
85	Relative theta power (full recording)	P	0.084	56
86	Asymmetry (62.5-125ms)	B	0.068	42
87	Range EEG (median, full recording)	P	0.062	49
88	Sharpness (4-8s)	B	0.058	0
89	EEG Envelope (median, full recording)	P	0.043	65
90	s_{min} (truncated power law fit to CDF of burst area)	B	-0.013	47
91	Relative theta power (quiet sleep)	P	-0.024	62
92	Range EEG (bandwidth)	O	-0.088	30
93	Burst duration[2] (95th percentile, quiet sleep)	P	-0.106	0
94	RMS burst duration[2] (quiet sleep)	P	-0.167	0
95	Burst number[1] (250-500ms)	B	-0.221	40
96	Intercept of linear fit to sharpness vs burst duration[1]	B	-0.310	4

References

[1] Roberts JA, Iyer KK, Finnigan S et al. Scale-free bursting in human cortex following hypoxia at birth. J Neurosci 2014; 34: 6557-6572.

[2] Palmu K, Stevenson N, Wikström S et al. Optimization of an NLEO-based algorithm for automated detection of spontaneous activity transients in early preterm EEG. Physiol Meas 2010; 31: N85-93.
